# Mortality in narcolepsy and other sleep clinic patients: a 25-year propensity-matched VA cohort study

**DOI:** 10.1007/s44470-026-00078-8

**Published:** 2026-05-04

**Authors:** Amir Sharafkhaneh, Yves Dauvilliers, Ahmed S. BaHammam, Mehrnaz Azarian, Michael Thorpy, Fang Han, Gulcin Benbir Senel, Murat Aksu, Javad Razjouyan

**Affiliations:** 1 Department of Medicine, Section of Pulmonary, Critical Care and Sleep Medicine, Baylor College of Medicine, 2002 Holcombe Blvd, Houston, TX 77030 USA; 2 Sleep Disorders and Research Center, Medical Care Line, Michael E. DeBakey VA Medical Center, Houston, TX USA; 3 Sleep-Wake Disorders Unit, Gui-de-Chauliac Hospital, CHU Montpellier, Institute of Neurosciences of Montpellier, University of Montpellier, Montpellier, France; 4 Department of Medicine, College of Medicine, University Sleep Disorders Center, King Saud University, Riyadh, Saudi Arabia; 5 Department of Medicine, Baylor College of Medicine, Houston, TX USA; 6 Center for Innovations in Quality, Effectiveness, and Safety, Michael E. DeBakey VA Medical Center, Houston, TX USA; 7 Albert Einstein College of Medicine, Bronx, NY USA; 8 Department of Sleep Medicine, Peking University People’s Hospital, Beijing, China; 9 Department of Neurology, Cerrahpasa Medical Faculty, Istanbul University-Cerrahpasa, Istanbul, Turkey; 10 Department of Neurology, Acibadem University, Istanbul, Turkey

**Keywords:** Hypersomnolence, Excessive daytime sleepiness, Hospitalization, Mortality, Narcolepsy

## Abstract

**Importance:**

Prior research shows increased mortality in patients with narcolepsy compared to the general population; overall, the evidence remains highly controversial and comparative data with general sleep clinic populations remains limited.

**Objective:**

We examined mortality and healthcare utilization among patients with narcolepsy compared to a general sleep clinic (GSC) cohort in the Veterans Affairs (VA) health system.

**Design:**

Retrospective cohort study.

**Setting:**

Nationwide Veterans Health Administration (VHA) data from October 1999 through March 2025.

**Participants:**

The study used relevant ICD-9/10 codes. We defined narcolepsy 1 (NT1) as patients with at least two NT1 ICD-9/10 codes. We constructed two propensity-matched (on age at index date, sex, race/ethnicity, and diagnosis year) comparison groups: (i) General Sleep Clinic (GSC; with no central disorders of hypersomnia ICD codes), matched 1:3 and (ii) other narcolepsy (ON) group as patients with at least two narcolepsy ICD-9/10 codes but no NT1-specific codes, matched 1:1 to NT1 group.

**Main outcomes and measures:**

The primary outcome was all-cause mortality. Secondary outcomes included all-cause hospitalization and emergency department/urgent care (ED/UC) visits. Adjusted odds ratios (aORs) and 95% confidence intervals (CIs) were estimated using multivariable logistic regression in the propensity score–matched cohorts, with GSC as the reference group and adjustment for age, body mass index, and Charlson Comorbidity Index.

**Results:**

The cohort included 4161 NT1, 4161 ON, and 12,843 GSC participants. The mean age differed in the groups (48.0, 47.6, and 43.8 years for NT1, ON, and GSC, respectively). All-cause mortality was more frequent in NT1 and ON compared to GSC (24.7%, 29.1%, and 15.0% in NT1, ON, and GSC, respectively). Compared with GSC, aOR was higher in NT1 (aOR 1.64, 95% CI 1.47–1.82) and ON (aOR 2.40, 95% CI 2.16–2.66). In contrast, increased aOR of all-cause hospitalization was observed only in NT1 (aOR 1.13, 95% CI 1.05–1.22) compared to GSC.

**Conclusions and relevance:**

Among veterans referred for sleep evaluation, adjusted OR of all-cause mortality was higher in narcolepsy patients compared to patients with other sleep disorders. The generalizability to non-VA populations remains uncertain. Future studies should identify cause-specific mortality and modifiable risk factors for prevention.

**Key points:**

**Question: **In veterans referred for sleep evaluation, is narcolepsy associated with higher all-cause mortality than general sleep clinic patients?

**Findings: **In a retrospective propensity-matched cohort study of VA data (1999–2025) including 4161 NT1, 4161 other narcolepsy, and 12,843 general sleep clinic participants, mortality was 24.7%, 29.1%, and 15.0%; adjusted odds of mortality were significantly higher for NT1 (aOR 1.64) and other narcolepsy (aOR 2.40) vs. general sleep clinic.

**Meaning: **These findings suggest elevated mortality risk in narcolepsy, highlighting the need for targeted prevention and risk-management strategies.

**Supplementary Information:**

The online version contains supplementary material available at 10.1007/s44470-026-00078-8.

## Introduction

Narcolepsy is a chronic neurologic sleep disorder characterized by excessive daytime sleepiness, dysregulated rapid eye movement (REM) sleep, and, in narcolepsy type 1 (NT1), cataplexy caused by orexin (hypocretin) deficiency [1]. It is a rare condition affecting 37.7/100,000 individuals in the United States (U.S.) population [2]. Symptoms of narcolepsy often begin in adolescence or early adulthood and persist throughout life, leading to significant functional impairment and reduced quality of life [3]. NT1 is defined by the presence of cataplexy and/or cerebrospinal fluid (CSF) orexin concentrations ≤ 110 pg/mL, reflecting marked hypothalamic orexin neuron loss. Narcolepsy type 2 (NT2), by contrast, is characterized by chronic sleepiness without cataplexy and typically normal or only mildly reduced CSF orexin levels. Because NT2 lacks a specific biomarker and overlaps clinically with other central disorders of hypersomnolence, accurate differentiation remains challenging in both research and clinical practice.

Prior epidemiologic investigations—using population registries, clinical cohorts, and insurance claims data—have attempted to evaluate differential risk profiles between NT1 and NT2, particularly with respect to cardiovascular disease (CVD) and mortality [4–6]. Although some studies suggest that NT1 may carry increased vulnerability to cardiometabolic and psychiatric dysregulation compared to NT2 [6, 7], these findings vary, and methodological limitations hinder definitive conclusions [8, 9]. In contrast to NT1, where orexin deficiency provides a plausible biologic link to adverse outcomes, the mechanisms underlying any potential increase in mortality among NT2 patients remain unclear. A critical challenge is disentangling the effects of the disorders themselves from the influence of long-term pharmacologic therapy [1]. Patients with NT1 are more likely to receive sodium oxybate, stimulants, and wake-promoting agents, all of which may impact cardiovascular risk and mortality estimates [10, 11].

While comparisons with the general population have suggested increased mortality in narcolepsy, such contrasts may exaggerate risk due to healthy-volunteer bias. Evaluating mortality relative to patients referred for sleep disorders provides a more pragmatic estimate of disorder-specific excess risk. Recent advances in causal epidemiology stress the importance of comparator group selection and bias mitigation in electronic health record studies [12, 13].

Current literature highlights the difficulty in distinguishing whether elevated mortality in narcolepsy reflects the underlying disease (particularly NT1) or consequences of chronic pharmacologic treatment [14]. Although modest increases in all-cause mortality have been observed in several cohorts [4–6], a recent nationwide Taiwanese study using both sibling and population-matched controls found no significant differences in all-cause or cause-specific mortality after adjustment for comorbidities [15]. These inconsistent findings underscore the need for studies that explicitly compare NT1, NT2, and other sleep-disordered patients within the same clinical system, rather than against general population samples.

Beyond mortality, large U.S. datasets indicate that narcolepsy is associated with substantial healthcare utilization and economic burden [16], yet most studies rely on administrative codes with limited phenotypic detail. Importantly, little is known about how mortality and acute healthcare use differ between NT1 and NT2, particularly within the U.S. Veteran population—a group with high comorbidity burdens and consistent access to integrated care.

The Veterans Health Administration (VHA) maintains a comprehensive electronic medical record (EMR) system used to care for millions of veterans. Researchers frequently use this EMR to address important clinical questions, and ICD codes have been validated for identifying clinical conditions. This database encompasses various domains including demographics, medications, comorbidities, healthcare utilization (outpatient and inpatient), laboratory data, procedures performed, and mortality [17].

We therefore hypothesized that NT1 and ON such as NT2 would each demonstrate increased all-cause mortality and acute healthcare utilization compared to other patients seen in sleep clinics, with NT1 exhibiting the highest risk. To test this, we used national VHA EMR data to compare mortality and healthcare utilization among patients with NT1, ON including NT2, and a general sleep clinic cohort.

## Methods

The study was approved by Baylor College of Medicine’s institutional review board (H-50294) and Michael E. DeBakey Veteran Affairs Medical Center research and development committee approved this research.

## Study design and cohort

**Fig. 1:**
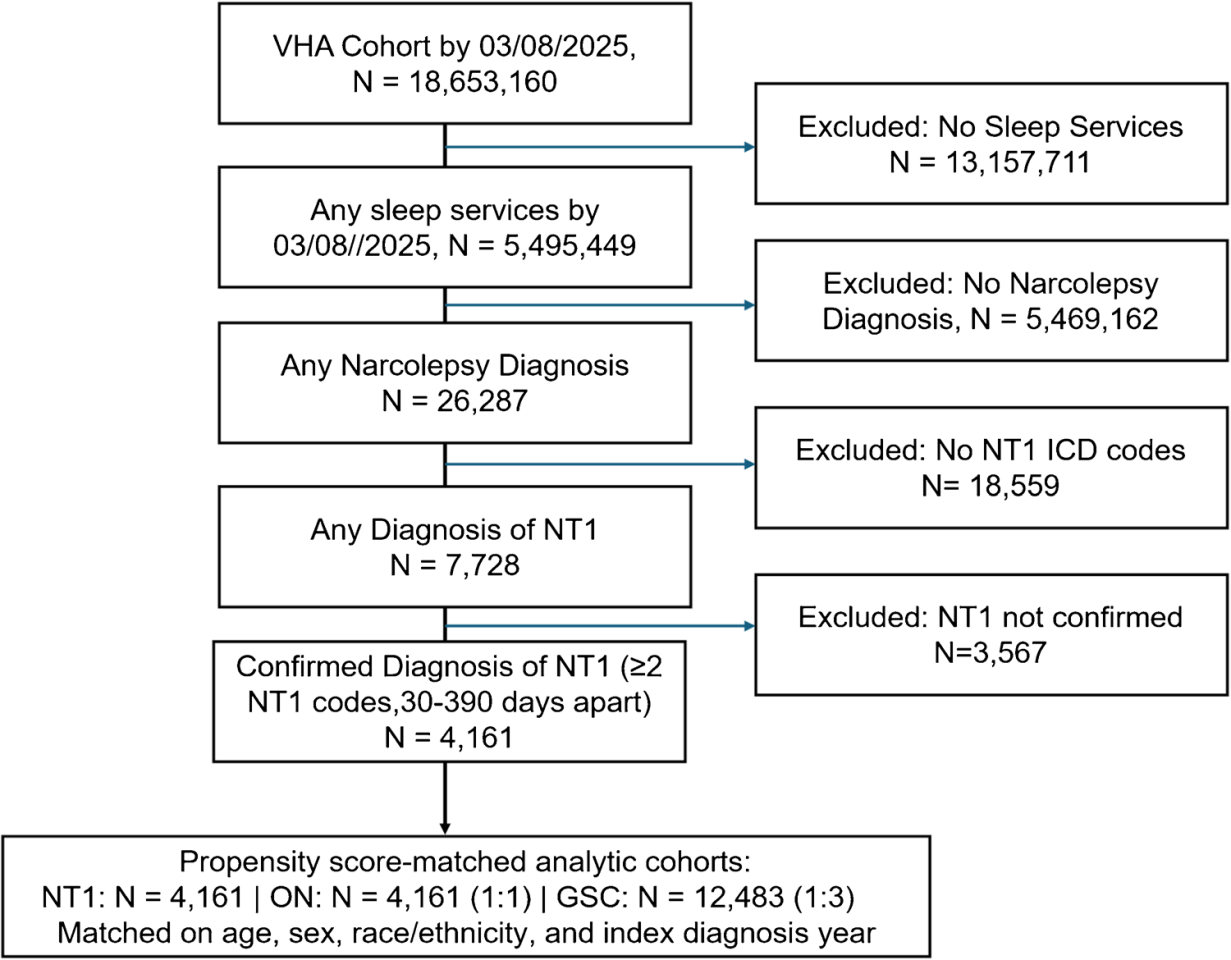
STROBE diagram for the population selection in this study

This is a retrospective cohort study with longitudinal follow-up using Veterans’ Health Administration (VHA) electronic medical records (EMRs). We used the STROBE statement for observational studies to report the cohort study. Figure 1 shows the initial cohort that included 5,495,449 veterans referred to the VA healthcare system from October 1, 1999, to March 8, 2025, with any International Classification of Diseases (ICD) 9th or 10th edition diagnostic codes or any sleep services based on Current Procedural Terminology (CPT) codes. Because PSG and MSLT data were not available in a standardized manner for cohort construction across the national VHA dataset, narcolepsy phenotypes were identified using ICD-9/10 computable phenotyping. For this study, the cohort was limited to individuals with any narcolepsy (ICD-9: “347X”; ICD-10: “G47.4X”). We further refined the cohort to those with narcolepsy 1 (NT1) based on the following ICD-9/10 codes (ICD-9: “347.01”, “347.11”; ICD-10: “G47.411”, “G47.421”). A previously validated algorithm was used to identify the confirmed diagnostic index date of each condition [18]. To improve specificity, a confirmed diagnosis required at least two relevant ICD-9/10 codes separated by at least 30 days within a 13-month window. In other words, in a sliding window of 13 months, if we observed two ICD codes with a minimum 30 days interval, we considered the diagnosis as confirmed. The first date-time that the diagnostic code appeared in the 13 months sliding window was considered as the index date.

## Study variables

### Outcome variables

The primary outcome of interest was all-cause mortality. Cause-specific mortality data were not available in this dataset. All-cause mortality data were gathered from the corporate data warehouse (CDW) VHA Vital Status table (sensitivity 98.3% and specificity 99.8% relative to the National Death Index) [19]. Secondary outcomes were all-cause hospitalization and emergency department or urgent care (ED/UC) encounters post-index date of diagnosis. We further refined these encounters to the 1 st year post-index date and the total period. We followed all the outcomes up to August 23, 2025.

### Exposure variable

Based on diagnosis codes, we defined three groups: (1) General Sleep Clinic (GSC), participants who used sleep services but had no ICD-9/10 codes for central disorders of hypersomnolence (most prevalent sleep disorder ICD-9/10 diagnoses are summarized in eTable1). The GSC cohort was not intended to represent a healthy control population. Rather, it was designed as a realistic clinical comparator; (2) narcolepsy type 1 (NT1), defined by ≥2 NT1 ICD-9/10 codes; and (3) other narcolepsy (ON), defined as patients with confirmed narcolepsy codes but no NT1-specific code to represent a heterogeneous category that could include NT2, unspecified narcolepsy, and potentially some misclassified cases. To control for nonmodifiable factors, we performed pairwise propensity score matching, matching GSC (1:3) and ON (1:1) to the NT1 cohort on age at index date, sex, race/ethnicity, and index diagnosis year. We matched both comparison groups to NT1 (rather than directly matching ON to GSC) because NT1 represented the most clinically specific narcolepsy phenotype and the GSC cohort was intentionally heterogeneous, making it less suitable as a stable matching anchor. However, this design is stronger for NT1-anchored comparisons and may leave residual confounding in the ON-versus-GSC comparison because those two groups were not directly propensity matched. We selected propensity score matching rather than inverse probability of treatment weighting (IPTW) because the cohort size allowed high-quality matching without substantial loss of power. Nearest-neighbor matching without replacement was performed using MatchIt (R v4.7.2), and covariate balance was assessed using standardized mean differences, with absolute SMD <0.10 considered acceptable.

### Other variables

We collected patient demographics such as age (categorized ≤ 40, 40–65, and ≥ 65 years.), body mass index (BMI) (categorized to obese with BMI ≥ 30 kg/m^2^), sex, race (White, Black, and others), ethnicity (Hispanic), and Charlson Comorbidity Index (CCI) at the index date. The CCI calculated inpatient and outpatient complications for an interval of 1 year before the index date [20]. We also reported the existence of insomnia and sleep apnea along with the following six groups of diseases: cardiovascular, metabolic, renal, pulmonary, neurologic, and psychiatric captured by ICD codes. The list of diagnoses and codes in each group is provided in eTable2.

### Medications

We extracted 7 groups of medications relevant to narcolepsy treatment as follows: (1) Anticataplectic 1 as Oxybate (various salts); (2) Anticataplectic 2 or antidepressants including Tricyclic Antidepressants (TCA), Serotonin-Norepinephrine Reuptake Inhibitor (SNRI), and Selective Serotonin Reuptake Inhibitor (SSRI); (3) Stimulants; (4) Wake-promoting agents; (5) Antipsychotics; and (6) Benzodiazepines. We reported the full list of medications in each group in eTable3. We considered each medication as confirmed if it was prescribed within a year prior to the index date of diagnosis, and the length of prescription was ≥30 days.

### Statistical analysis

Descriptive statistics are computed for all variables by study group, including means with standard deviations for continuous variables and frequencies with proportions for categorical variables. Baseline differences between groups are compared using two-tailed independent t-tests (means) and chi-square tests (percentages) to evaluate characteristics in each group. Significant variables are included as covariates in multivariate regression analysis. Outcomes were analyzed as binary endpoints (occurrence of each outcome during follow-up), and odds ratios (ORs) with 95% confidence intervals (95% CIs) were estimated using unconditional multivariable logistic regression in the propensity score–matched cohorts, with GSC as the reference group. Models were adjusted for age, body mass index (categorized as <18.5, 18.5–25, 25–30 [reference], 30–35, 35–40, and ≥40), and Charlson Comorbidity Index (CCI). Logistic regression was selected over time-to-event methods (e.g., Cox models) to provide a consistent framework across mortality and healthcare utilization outcomes, where event timing and recurrent utilization patterns are not uniformly modeled in this dataset. A *p*-value <0.05 was considered statistically significant. Cohorts were curated using Structured Query Language (SQL), and analyses were conducted in R (RStudio).

## Results

The final study population included 4161 individuals with NT1, 4161 with ON, and 12,483 propensity-matched participants in the general sleep clinic (GSC) cohort consisting primarily of patients with OSA (65.4%) and insomnia (26.6%). Table 1 summarizes demographic characteristics. All three cohorts were predominantly male (73.8% in GSC, 82.2% in NT1, and 81.2% in ON). The GSC group had a slightly higher proportion of females. The mean age of the narcolepsy cohorts was in the late 40 s, reflecting the demographic structure of the veteran population rather than the typical age at symptom onset described in community-based narcolepsy samples. The GSC cohort was approximately 5 years younger (mean age 43.8 ± 15.6 in GSC, 48 ± 16.6 in NT1, and 47.6 ± 16.0 in ON). The mean BMI across all groups was in the obese range (32.7 ± 6.4 in GSC, 30 ± 5.9 in NT1, and 30.4 ± 6.3 in ON). The racial and ethnic distribution was similar among cohorts, with most participants identifying as White, followed by Black, and a smaller proportion identifying as Hispanic or other races.

**Table 1:** Demographic characteristics of propensity-matched narcolepsy and general sleep clinic cohorts

Propensity-matched cohorts	GSC	NT1	ON
*N*	12,483	4161	4161
Male sex: *N *(%)	9215 (73.8)	3421 (82.2)	3378 (81.2)
INDEX_DX_ Year < 2005: *N *(%)	575 (4.6)	1231 (29.6)	1920 (46.1)
INDEX_DX_ Year ≥ 2005 < 2014: *N *(%)	6438 (51.6)	1175 (28.2)	1 (0)
INDEX_DX_ Year ≥ 2014: *N *(%)	5470 (43.8)	1755 (42.2)	2240 (53.8)
Age: Mean (SD)	43.8 (15.6)	48 (16.6)	47.6 (16.0)
BMI: Mean (SD)	32.7 (6.4)	30 (5.9)	30.4 (6.3)
Race/ethnicity			
White, *N *(%)	6410 (51.3)	2333 (56.1)	2185 (52.5)
Black, *N *(%)	2512 (20.1)	890 (21.4)	915 (22.0)
Hispanic, *N *(%)	908 (7.3)	230 (5.5)	246 (5.9)
Native American, *N* (%)	144 (1.2)	038 (0.9)	25 (0.6)
Asian, *N *(%)	443 (3.6)	056 (1.3)	44 (1.1)
Unknown, *N* (%)	2066 (16.6)	614 (14.8)	746 (17.9)

*INDEX*_*DX*_, Index Diagnostic Date, *GSC *general sleep cohort, participants who used sleep services but with no diagnosis of central hypersomnia disorders, *NT1*, Computably confirmed Narcolepsy type 1, *ON*, Computably confirmed narcolepsy with no NT1 ICD codes

GSC and ON are propensity matched to NT1 cohort based on age, sex, race/ethnicity, and year of index diagnosis

Table 2 shows the comorbidity burden and the prevalence of specific conditions at the index date. The proportion of patients with Charlson Comorbidity Index (CCI) ≥ 2 was similar across the three groups. Compared to the GSC cohort, both narcolepsy groups had a lower prevalence of cardiovascular (47.7% in GSC vs. 41.8% in NT1 and 43.9% in ON), metabolic (44.5% in GSC vs. 35.7% in NT1 and 37.5% in ON), pulmonary (67.6% in GSC vs. 63.9% in NT1 and 44.5% in ON), psychiatric disorders (53.4% in GSC vs. 43.0% in NT1 and 48.0% in ON), obstructive sleep apnea (OSA) (65.4% in GSC vs. 33.2% in NT1 and 36.7% in ON), and insomnia (26.6% in GSC vs. 15.0% in NT1 and 18.0% in ON).

**Table 2:** Comorbidity burden and clinical characteristics of propensity-matched narcolepsy and general sleep clinic cohorts

	GSC	NT1	ON
N	12,483	4161	4161
CCI ≥ 2: *N* (%)	1735 (13.9)	488 (11.7)	574 (13.8)
Cardiovascular^a^, *N* (%)	5954 (47.7)	1739 (41.8)	1828 (43.9)
Metabolic^b^,* N* (%)	5558 (44.5)	1484 (35.7)	1560 (37.5)
Renal^c^, *N* (%)	1119 (9.0)	765 (15.2)	340 (8.2)
Pulmonary^d^, *N *(%)	8434 (67.6)	3217 (63.9)	1853 (44.5)
OSA, *N* (%)	8159 (65.4)	1381 (33.2)	1526 (36.7)
Insomnia: *N* (%)	3322 (26.6)	623 (15.0)	750 (18.0)
Neurologic^e^, *N* (%)	2171 (17.4)	778 (18.7)	863 (20.7)
Psychiatric^f^, *N* (%)	6661 (53.4)	1789 (43.0)	1999 (48.0)

*GSC*, general sleep cohort, participants who used sleep services but with no diagnosis of any hypersomnia disorder, *NT1*, Computably confirmed Narcolepsy type 1, *ON*, Computably confirmed narcolepsy with no NT1 ICD codes, GSC and ON are matched to NT1 cohort on age, sex, race/ethnicity, and year of index diagnosis; *CCI*, Charlson Comorbidity Index

^a^Any of the following: hypertension, heart disease, other and ill-defined heart disease, cardiovascular disease including hypertension, coronary atherosclerosis and other heart disease, heart failure, peripheral artery disease, congestive heart failure, arrhythmia, cardiomyopathy, acute cardiac injury, acute myocardial infarction

^b^Any of the following: hyperlipidemia, diabetes, diabetes type 2, diabetes without/with complication

^c^Any of the following: kidney disease, chronic kidney disease, nephrosis, urinary stone, acute kidney failure, chronic kidney failure, dialysis

^d^Any of the following: obstructive sleep apnea, chronic lung disease, chronic obstructive pulmonary disease, smoking, dyspnea, cough, bronchitis, asthma, acute respiratory failure, pulmonary heart disease, lower respiratory infection, venous thromboembolism, acute respiratory distress syndrome, emphysema, pulmonary embolism

^e^Any of the following: cerebrovascular disease, chronic neurological disease, ischemic stroke, headache, myalgia, epilepsy, Parkinson, Alzheimer

^f^Any of the following: major depressive disorder, anxiety, PTSD, alcohol dependence, drug dependence, bipolar, schizophrenia

Full list of comorbidities in eTable 2

Medication prescription patterns are presented in Table 3. In the NT1 cohort, 91.7% of patients were prescribed at least one narcolepsy medication, compared with 77.1% in the ON cohort. Oxybate prescription (anticataplectic G1) was observed in 14.3% of NT1 patients and 1.3% of ON patients. 69.6% of NT1 patients and 58.3% of ON patients were prescribed at least 1 Anticataplectic 2 including TCAs, SSRIs, and SNRIs. Stimulants were prescribed for 52.0% of NT1 and 33.8% of ON patients. Wake-promoting agents were prescribed for 64.4% and 39.4% of NT1 and ON patients, respectively. Pitolisant and Solriamfetol were prescribed infrequently. Supplemental eTable3 provides a detailed list of narcolepsy-related medications.

**Table 3:** Medications prescribed to narcolepsy type 1 and non-type 1 cohorts

	NT1	ON
*N*	4161	4161
Any narcolepsy medication^*^	3816 (91.7)	3210 (77.1)
Anticataplectic G1 (all oxybate): *N* (%)	594 (14.3)	56 (1.3)
Any anticataplectic G1 (sodium oxybate): *N* (%)	487 (11.7)	37 (0.9)
At least 1 anticataplectic G2: *N* (%)	2894 (69.6)	2426 (58.3)
At least 2 anticataplectic G2: *N* (%)	920 (22.1)	895 (21.5)
Anticataplectic G2 (TCA only): *N* (%)	2593 (62.3)	2051 (49.3)
Anticataplectic G2 (SNRI only): *N* (%)	229 (5.5)	291 (7.0)
Anticataplectic G2 (SSRI only): *N* (%)	1065 (25.6)	1069 (25.7)
Stimulants: *N *(%)	2165 (52.0)	1408 (33.8)
Any wake-promoting agents: *N* (%)	2681 (64.4)	1639 (39.4)
Antipsychotics: *N* (%)	300 (7.2)	366 (8.8)
Benzodiazepines:* N* (%)	542 (13.0)	655 (15.7)

*NT1*, Computably confirmed Narcolepsy type 1, *ON*, Computably confirmed narcolepsy with no NT1 ICD codes; GSC and ON are matched to NT1 cohort on age, sex, race/ethnicity, and year of index diagnosis

^*^Including Anticataplectics 1 and 2, Stimulants, and Wake promoting agents

See Table 2 Supplemental for list of medications in each class

Health outcomes and utilization are shown in Table 4. All-cause mortality proportions were 15.0%, 24.7%, and 29.1% in the GSC, NT1, and ON cohorts, respectively. All-cause hospitalization frequency (both within the first year and during the entire follow-up period) was higher among narcolepsy patients compared to GSC participants. In contrast, emergency department or urgent care (ED/UC) visits were more frequent in the GSC group compared to both narcolepsy cohorts.

**Table 4:** Odds ratios for mortality and healthcare utilization outcomes across narcolepsy and general sleep clinic cohorts

	Event Numbers (%)	Raw OR (95% CI)	Adj OR^*^ (95% CI)
All-cause mortality			
* GSC*	1868 (15)	Reference	Reference
* NT1*	1027 (24.7)	1.86 (1.71, 2.03)	1.64 (1.47, 1.82)
* ON*	1211 (29.1)	2.33 (2.15, 2.53)	2.40 (2.16, 2.66)
1st year all-cause hospitalization from the index date			
* GSC*	1192 (9.6)	Reference	Reference
* NT1*	859 (17.1)	0.93 (0.82, 1.05)	0.91 (0.80, 1.03)
* ON*	523 (12.6)	1.36 (1.22, 1.52)	1.31 (1.17, 1.46)
Total all-cause hospitalizations			
* GSC*	4379 (35.1)	Reference	Reference
* NT1*	2173 (43.1)	1.16 (1.08, 1.25)	1.13 (1.05, 1.22)
* ON*	1524 (36.6)	1.07 (0.99, 1.15)	1.03 (0.95, 1.11)
1st year ED/UC visits from the index date			
* GSC*	3471 (27.8)	Reference	Reference
* NT1*	1123 (22.3)	0.62 (0.57, 0.68)	0.67 (0.61, 0.73)
* ON*	583 (14.0)	0.42 (0.38, 0.47)	0.44 (0.40, 0.49)
Total ED/UC visits			
* GSC*	8133 (65.2)	Reference	Reference
* NT1*	2844 (56.5)	0.94 (0.87, 1.01)	1.01 (0.94, 1.09)
* ON*	2200 (52.9)	0.6 (0.56, 0.64)	0.63 (0.59, 0.68)

*GSC*, general sleep cohort, participants who used sleep services but with no diagnosis of any hypersomnia disorder; *NT1*, Computably confirmed Narcolepsy type 1, *ON*, Computably confirmed narcolepsy with no NT1 ICD codes, GSC and ON are matched to NT1 cohort on age, sex, race/ethnicity, and year of index diagnosis

^*^ORs are adjusted for age, BMI, and CCI

Unadjusted analyses demonstrated significantly higher odds of mortality among both narcolepsy groups compared to the GSC as the reference cohort. Total all-cause hospitalization risk remained elevated for both narcolepsy cohorts, whereas 1 st year ED/UC visits were significantly lower. Compared with the GSC cohort, both NT1 and ON were associated with higher adjusted odds of all-cause mortality (adjusted for age, BMI and comorbidity burden). However, because ON and GSC were not directly propensity-matched to one another, the ON-versus-GSC comparison should be interpreted with greater caution than the NT1-anchored comparisons. These adjusted odds ratios (aORs) for mortality were 1.64 (95% CI 1.47–1.82) for NT1 and 2.40 (95% CI 2.16–2.66) for ON. Adjusted ORs of Total all-cause hospitalization were 1.13 (95% CI 1.05, 1.22) for NT1 and 1.03 (95% CI 0.95, 1.11) for ON, and aORs of 1 st year ED/UC visits were 0.67 (95% CI 0.61, 0.73) and 0.44 (95% CI 0.40, 0.49), respectively.

## Discussion

We analyzed data from three propensity-matched cohorts—general sleep clinic (GSC), narcolepsy type 1 (NT1), and ON (including NT2)—derived from the Veterans Health Administration (VHA) electronic medical records over a 25-year period. The study population was predominantly male and demonstrated a high prevalence of multiple comorbid conditions. Both narcolepsy groups were treated with combinations of medications commonly used for symptom control. Importantly, our findings demonstrated, for the first time, significantly higher all-cause mortality among patients with NT1 and ON compared with the GSC cohort, even though the GSC cohort was younger and exhibited a higher prevalence of nearly all comorbid conditions. The associations persisted even after adjustment for age, body mass index (BMI), and Charlson Comorbidity Index (CCI). All-cause hospitalization prevalence was elevated in the narcolepsy cohorts, whereas emergency department and urgent care visits were less frequent compared with the GSC group.

The results of the association between narcolepsy and mortality compared to the general population as the control group are debated. Ohayon et al. reported that the narcolepsy population had an approximate 1.5-fold excess mortality relative to those without narcolepsy [5], while Jennum et al. reported that the mortality rate due to narcolepsy was slightly but not significantly higher [6]. Another retrospective study in Taiwan showed that narcolepsy was not associated with excess all-cause or cause-specific mortality [15]. In contrast, other studies reported an increased risk of cardiovascular events in narcolepsy patients compared to control groups [9, 21, 22]. Unlike these prior investigations, our study directly compared narcolepsy patients to other sleep-disordered patients rather than population controls, providing a more clinically relevant perspective on mortality risk. Notably, while prior reports attributed elevated mortality in narcolepsy in part to comorbidities, our findings reveal higher mortality in narcolepsy even when the comparator group—GSC—exhibited a greater burden of cardiovascular, metabolic, pulmonary, and psychiatric conditions.

The comorbidity profiles in our cohorts reflect both known patterns and unique characteristics of the VHA population like higher prevalence of cardiovascular disorders [23]. Cardiovascular and metabolic conditions, psychiatric disorders, and sleep-related comorbidities were all highly prevalent across our cohorts, consistent with previous literature [6, 9, 22, 24]. However, unlike population-based studies, our GSC cohort—representing sleep clinic patients (excluding those with central and idiopathic hypersomnia disorders)—had a higher prevalence of almost all major comorbidities, including cardiovascular, metabolic, and psychiatric conditions. Despite this elevated comorbidity burden and the younger age in the GSC group, narcolepsy patients still experienced higher mortality and hospitalization rates, underscoring that the excess risk in narcolepsy is independent of comorbidity burden. The higher risk may reflect disease-specific factors, treatment-related effects, or both. The disease-specific risks include cardiovascular, behavioral, medication-related, and accident-related mechanisms reported in prior literature. Orexin deficiency in NT1 provides a biologically plausible mechanism for adverse outcomes. Because medication exposure was summarized descriptively rather than modeled as a time-varying causal factor, and because biomarker data such as CSF orexin were unavailable, we could not determine whether excess mortality was driven primarily by narcolepsy neurobiology, pharmacotherapy, or their interaction. Further, the present study cannot distinguish among these possibilities because cause-specific death data were unavailable.

A notable feature of this analysis is the use of a general sleep clinic cohort as a pragmatic clinical comparator rather than a general population control. This comparator was not healthy; it included high rates of OSA, insomnia, and other comorbid conditions. Accordingly, this design may provide a more conservative estimate of excess risk associated with narcolepsy than studies using population controls, but it may also attenuate the apparent magnitude of narcolepsy-specific risk. Our findings should therefore be interpreted as demonstrating excess mortality relative to a clinically complex sleep-clinic population, rather than relative to a healthy baseline population. Interestingly, this control group exhibited a higher burden of comorbidities such as cardiovascular disease, diabetes, and psychiatric disorders, yet still had lower adjusted mortality. This raises a fundamental question: does narcolepsy confer mortality risk through mechanisms that are independent of, or synergistic with, comorbidity load? The disproportionate risk may reflect disease-specific factors such as orexin deficiency, medication side effects, or delayed recognition and care coordination. This contrasts with prior studies and highlights that the pathophysiology of narcolepsy and possibly disease-specific factors, including excessive daytime sleepiness (EDS) and reduced quality of life and medication exposure, may contribute directly to adverse outcomes. Previous studies showed the independent association between EDS and all-cause mortality in individuals older than 40 [25–27]. Furthermore, recent data showed the probable protective effect of mild to moderate levels of sleep apnea [28], which might also justify some parts of the lower mortality risk in GSC compared to narcolepsy.

Medication prescription patterns in our study revealed frequent use of anticataplectic agents, stimulants, and wake-promoting medications, particularly in NT1 patients. While these medications are critical for symptom management, their pharmacologic profiles and potential cardiovascular effects may partially contribute to increased mortality and hospitalization. Previous studies highlighted that commonly prescribed narcolepsy treatments may pose cardiovascular risks and necessitate specific precautions [9]. For instance, a retrospective study found that a higher proportion of patients using modafinil required initiation or escalation of antihypertensive therapy (2.4%) compared with those receiving placebo (0.7%), underscoring the need for closer cardiovascular monitoring [29]. Similarly, individuals with NT1 treated with WPA experienced elevated heart rate and blood pressure relative to untreated patients, with these effects being more pronounced when WPA were used in combination with anticataplectic agents [30]. Conversely, the lower rates of emergency department and urgent care visits in narcolepsy cohorts should be interpreted cautiously. This pattern could reflect greater reliance on better continuity of care, adherence to prescribed regimens, or reliance on scheduled outpatient management. The longitudinal and integrated nature of the VA system likely amplified medication exposure compared with community-based cohorts. However, alternative explanations include barriers to acute-care access, under-recognition of emergency symptoms, differences in care-seeking behavior, or incomplete capture of non-VA emergency utilization.

Although the associations persisted after adjustment for major comorbidities, age, and BMI, the possibility of residual confounding and unmeasured behavioral determinants (physical activity, smoking intensity, adherence to therapy) cannot be ruled out. The Veterans Affairs Corporate Data Warehouse has demonstrated high accuracy for mortality ascertainment, with validation studies showing that VA death registries combined with inpatient records are as accurate and more up to date than the National Death Index [31]. Given this near-complete vital status accuracy in VA datasets, misclassification bias is unlikely to drive our findings. Some formulations like low-sodium oxybate may offer improved cardiovascular safety profiles compared to traditional sodium oxybate [32] Future mechanistic studies should integrate real-world pharmacotherapy data to model causal mediation pathways [33].

From a causal perspective, the observed association between NT1 and adverse outcomes likely reflects both direct disease-related mechanisms (orexin deficiency–linked autonomic dysregulation) and treatment-mediated pathways. Future studies are warranted to apply modern g-methods, including target trial emulation and inverse probability weighting, which may help disentangle these effects in subsequent analyses [34].

Our study has several strengths, including a large sample size, a long follow-up duration spanning more than two decades, a novel comparison of narcolepsy with other sleep clinic patients, diverse racial/ethnic representation, comprehensive comorbidity assessment, and detailed medication data. Further, although the definition and treatment of narcolepsy have evolved over time, particularly after 2014, our propensity-matched score matching included the index diagnostic date, ensuring temporal comparability across cohorts and supporting the consistency of our results across different diagnostic periods.

The main limitation is lack of cause-of-death and cause of hospitalization data which limits our knowledge about deaths due to car crashes, as Pizza et al. demonstrated how patients affected with central disorders of hypersomnolence had increased risk of recent car crashes compared to subjects from the general population and this would potentially be reversed by long-term treatment [35]. An important limitation of this study is the lack of cause-specific death and hospitalization data. As a result, we could not determine whether the observed excess mortality was driven by cardiovascular events, motor vehicle accidents, psychiatric causes, medication-related complications, or other mechanisms. Therefore, these findings should be interpreted as evidence of increased overall mortality risk rather than evidence for any specific causal pathway or intervention target. Future studies linking VHA data to cause-of-death records and diagnosis-specific hospitalization data will be necessary to define actionable prevention strategies. Despite the absence of cause-of-death data in this VHA dataset, the mortality signal observed aligns with literature suggesting elevated cardiovascular and accidental death risks in narcolepsy. For example, prior work shows increased risk of motor vehicle accidents and cardiovascular morbidity in patients with narcolepsy treated with stimulants [36, 37] The other limitations include absence of medication dosing information, and incomplete capture of healthcare utilization outside the VHA system. An important limitation of our study is the cohort assignment. We relied on administrative ICD-9/10 codes rather than gold-standard diagnostic measures such as PSG followed by MSLT. To mitigate this limitation, we improved diagnostic certainty by requiring two narcolepsy-related codes recorded 30–390 days apart using a validated computable phenotyping approach. This algorithm aligns with validated computable phenotyping approaches for narcolepsy developed for EHR-based research [38]. However, we acknowledge that some wrong assignment may remain. This concern is particularly relevant for the ON group, which was intentionally broad and may include NT2, unspecified narcolepsy, and other heterogeneous or misclassified hypersomnolence presentations. Thus, findings for ON should not be interpreted as applying to a pure NT2 cohort. To avoid immortal time bias, follow-up time accrued before confirmation of the narcolepsy diagnosis was excluded, and the index date was defined as the earliest confirmed diagnosis fulfilling the algorithmic validation criteria (two ICD codes, ≥ 30-day interval) [39]. Furthermore, some misclassification may remain due to inclusion of unspecified narcolepsy codes in ON, and because GSC and ON were matched to NT1 (not each other), residual confounding may persist for ON vs. GSC comparisons.

Several factors limit the external validity of the study findings. First, the VA cohort was predominantly male (73.8%–82.2%), whereas non-VA narcolepsy populations include a greater proportion of women. Second, the mean age at index diagnosis in our cohorts was in the mid-to-late 40 s, which may differ from the younger age at symptom onset or diagnosis often observed outside the VA system. Third, veterans carried a higher burden of cardiovascular, metabolic, pulmonary, and psychiatric comorbidity, and care within the integrated VHA system may influence diagnosis, treatment patterns, and outcome ascertainment in ways that differ from community practice. Accordingly, these results should be interpreted primarily as applicable to veterans receiving sleep care within the VA and should be validated in younger, more demographically representative civilian cohorts. Finally, incomplete capture of healthcare utilization outside the VHA system is an additional limitation, particularly for emergency department and urgent care outcomes. Therefore, lower observed ED/UC utilization in narcolepsy cohorts may not necessarily indicate fewer acute clinical events or better continuity of care.

The persistence of excess mortality among patients with narcolepsy, despite having a lower comorbidity burden than the general sleep clinic cohort, underscores the importance of better understanding the effects of lack of orexin on autonomic activation which can be accomplished by incorporating longitudinal biomarker and wearable device data.

In conclusion, after adjusting for comorbidity burden, age, and BMI, narcolepsy patients exhibited higher all-cause mortality and acute healthcare utilization compared with a propensity-matched sleep clinic cohort with no central hypersomnolence disorders. Notably, these findings persisted despite the control cohort exhibiting higher rates of comorbid conditions, highlighting an excess mortality signal among patients with narcolepsy relative to a clinically complex sleep-clinic comparator. Future studies should focus on identifying the causes of death and hospitalization and evaluating the long-term impact of medications in narcolepsy patients.

## Supplementary Information

Below is the link to the electronic supplementary material.

ESM 1 (DOCX 35.5 KB)

## Data Availability

The datasets generated and/or analyzed during the current study are not publicly available due to U.S. Department of Veterans Affairs data use regulations but are available from the corresponding author upon reasonable request and with appropriate VA approvals.
